# Improved forearm rotation even after early conversion to below-elbow cast for non-reduced diaphyseal both-bones forearm fractures in children: a secondary 7.5-year follow up of a randomized trial

**DOI:** 10.2340/17453674.2023.18340

**Published:** 2023-10-06

**Authors:** Linde MUSTERS, Leon W DIEDERIX, Pim P EDOMSKIS, Kasper C ROTH, Joyce L BENNER, Gerald A KRAAN, Jan H ALLEMA, Max REIJMAN, Denise EYGENDAAL, Joost W COLARIS

**Affiliations:** 1 Department of Orthopaedics and Sports Medicine, Erasmus MC, University Medical Centre Rotterdam; 2 Department of Orthopedics, Elkerliek Hospital, Helmond; 3 Department of Surgery, Erasmus MC, University Medical Centre Rotterdam; 4 Department of Orthopaedic Surgery, Centre for Orthopaedic Research Alkmaar (CORAL), Northwest Clinics, Alkmaar; 5 Department of Orthopedics, Reinier Haga Orthopedisch Centrum, Zoetermeer; 6 Department of Surgery, Haga Hospital, The Hague, the Netherlands

## Abstract

**Background and purpose:**

previous RCT compared short-term results of above-elbow cast (AEC) with early conversion to below-elbow cast (BEC) in children with non-reduced diaphyseal both-bone forearm fractures. After 7 months both groups had comparable function. Our primary aim was to investigate whether forearm rotation improves or worsens over time. Secondary aims were loss of flexion and extension of the elbow and wrist, patient-reported outcomes measures, grip strength ratio, and radiographic assessment.

**Patients and methods:**

We performed long-term follow-up (FU) of a previous RCT. All patients were invited again for the long-term FU measurements. Primary outcome was limitation of forearm rotation. Secondary outcomes were loss of flexion and extension of the elbow and wrist compared with the contralateral forearm, the ABILHAND-Kids questionnaire and the Disabilities of the Arm, Shoulder and Hand (DASH) questionnaire, grip strength ratio, and radiographic assessment.

**Results:**

The mean FU was 7.5 (4.4–9.6) years. Of the initial 47 children, 38 (81%) participated. Rotation improved in both groups over time, with no significant difference in the final forearm rotation: 8° (SD 22) for the AEC group and 8° (SD 15) for the BEC group with a mean difference of 0° (95% confidence interval –13 to 12). Secondary outcomes showed no statistically significant differences. Finally, children < 9 years almost all have full recovery of function.

**Conclusion:**

Long-term follow-up showed that loss of forearm rotation after a non-reduced diaphyseal both-bone forearm fracture improved significantly compared with that at 7 months, independent of the initial treatment and children aged < 9 will have almost full recovery of function. This substantiates that the remaining growth behaves like a “friend” at long-term follow-up.

Forearm fractures of both bones account for 40% of all pediatric fractures [1]. They are more common in boys and 20% of these fractures are located in the diaphysis. Usually, non-reduced diaphyseal forearm fractures of both bones are treated with an above-elbow cast (AEC) for 6 weeks, and in selective cases converted to below-elbow cast (BEC) for the last weeks. In case of instability of (1 of) the fractures after reduction, intramedullary nails or plates are advocated to stabilize the fracture(s) [2].

Previous studies showed a persisting loss of forearm rotation of up to 15% of the normal range of motion, especially in diaphyseal located fractures [3]. The underlying mechanism seems to be malunion of the radius and/or the ulna, combined with contractures of soft tissue such as the interosseous membrane [4]. This leads to the suggestion made by Colaris et al. that early conversion to BEC in the treatment of children with non-reduced diaphyseal forearm fractures of both bones could potentially result in fewer contractures of the interosseous membrane. Furthermore, this group also shows less remaining loss of forearm rotation 18° (SD 17) compared with 23° (SD 22) in the AEC group, but this was not statistically significant. This short-term follow-up showed positive results, but long-term follow-up was missing [5]. Previous studies have already demonstrated a relationship between age and the ability to correct bony deformities. Although both groups already showed good results in function at short-term follow-up, it is interesting to see whether remaining growth in these 2 groups behaves like a “friend or an enemy.” We aimed to evaluate the results of these 2 treatment groups. Our focus was on the primary outcome, loss of forearm rotation. Secondary outcome measures were loss of flexion and extension of the elbow and wrist, patient-reported outcomes measures (PROMS: ABIL-HAND-Kids questionnaire and quick DASH [Disabilities of the Arm, Shoulder, and Hand] questionnaire), grip strength ratio, and radiographic assessment [6].

## Patients and methods

Our study is a long-term follow-up of a previous RCT. All 47 patients who had been previously included in the RCT by Colaris et al. [5] between January 2006 and August 2010 were invited to visit the outpatient clinic for additional clinical and radiographic assessment. This population consisted of patients who were included after they visited the emergency department of 1 of 4 participating hospitals: Erasmus Medical Center (Rotterdam), HAGA Hospital (The Hague), Reinier de Graaf Hospital (Delft), and Franciscus Gasthuis and Vlietland Hospital (Rotterdam). Inclusion criteria were all children < 16 with diaphyseal both-bone forearm fracture with no indication for reduction, based on predefined criteria (Table 1). These children had been randomized for the previous study to treatment with 6 weeks of above-elbow cast (AEC) or early conversion to below-elbow cast (BEC), which means 3 weeks of above-elbow cast followed by 3 weeks of below-elbow cast. All patients received allocated treatment. The minimum follow-up was set at 5 years. This study complies with the CONSORT statement.

**Table 1:** Criteria for reduction of the fracture of radius and/or ulna based on anteroposterior and/or lateral radiographs

Type of displacement	Age in years	Displacement
Angulation	< 10	> 10°
	10–16	> 15°
Translation	< 16	> half of bone diameter
Rotation	< 16	> 0°

### Outcome measures

Our primary outcome was change in loss of forearm rotation in comparison with the contralateral side and a comparison between the 2 casting methods. Secondary outcome measures were change in loss of flexion and extension of the elbow and wrist compared with the contralateral forearm, the Dutch version of the DASH and ABILHAND-Kids questionnaire, grip strength, and radiographic assessment of the radius and ulna. Cosmetics was measured as parents/child VAS 0–10 and surgeon VAS 0–10 (10 being the optimal score). An independent orthopedic surgeon (LD) measured the forearm rotation, flexion, and extension of the elbow and wrist in a standardized manner with the use of a goniometer. Before each measurement, the examiner ensured that the child was standing in an upright position. Meanwhile, the elbow was positioned firmly against the torso to eliminate compensating forearm rotation by using movements of the elbow and shoulder. The elbow was flexed at 90° with the forearm in mid-position and the wrist in neutral. Grip strength was determined using a JAMAR dynamometer (JLW Instruments, Chicago, IL, USA) on both sides and calculating a ratio of grip strength of the affected forearm/contralateral side. Furthermore, patients were asked to fill in the DASH and the ABILHAND-Kids questionnaires. The radiographic assessment included measurement of the coronal and sagittal angulation of the radius and ulna conducted by 1 of the authors (PE). Analyses were done using locally available analysis programs such as PACS and JiveX.

To rule out potential effects of attrition we undertook a representability analysis. The included population was compared with the loss to follow-up population. This analysis showed no significant difference in age, sex, forearm rotation, or secondary outcomes such as ABILHAND-Kids questionnaire, VAS, and complication rate, concluding that the follow-up group was representative of the whole original study group (Table 2).

**Table 2:** Representability of the lost to follow-up and included population. Values are mean (SD) unless otherwise stated

Factor	Lost to FU	Included	Mean difference (CI)
Number of patients	9	38	/
Age at trauma	6.2	6.4	–0.3 (–2.8 to 2.3)
range	(1.8–12.3)	(0.9–14.9)	/
Male sex, n	5	18	/
Forearm rotation at 7 months, °	135 (30)	130 (26)	4.6 (–15 to 25)
Loss of forearm rotation, °	19 (22)	21 (19)	–2.2 (–20 to 15)
ABILHAND-Kids score ^a^	42 (0)	41 (7.8)	1.5 (–5.7 to 8.7)
VAS-cosmetics score ^b^			
parents/child	7.4 (2.7)	7.9 (2.6)	–0.5 (–2.5 to 1.5)
surgeon	8.5 (1.3)	7.9 (2.2)	0.6 (–0.9 to 2.2)
Complications, %	0.2 (0.4)	0.4 (0.5)	–0.1 (–0.5 to 0.2)

CI = 95% confidence interval.

a ABILHAND-Kids questionnaire score 0–42. 42 is optimal score.

b VAS score 0–10. 10 is optimal score.

### Statistics

We compared the baseline characteristics and functional outcome at 7 months and at 7.5 years between the included patients and those lost to follow-up to rule out potential effects of attrition. Long-term results of primary and secondary outcomes were compared between the 2 groups (AEC vs. BEC) using independent T-testing and crosstabs. Results are presented as mean with 95% confidence interval (CI) and standard deviation (SD) because of normal distribution. Furthermore, to address the issue of missing data, linear mixed-model analyses were done for the primary outcome to compare the between-group differences over the different time points where means with 95% CI are presented.

We performed a subgroups analysis for both groups on loss of forearm rotation. The pronation and supination were scaled using the grading system as described by Daruwalla with excellent, good, fair, and poor results for, respectively, 0–10°, 11–20°, 21–30°, and ≥ 31° of limitation [7]. All statistical analyses were performed using IBM SPSS Statistics version 23 (IBM Corp, Armonk, NY, USA).

### Ethics, registration, consent, funding, and disclosures

For this post-trial follow-up study ethics approval was again obtained from the regional medical ethical committee (Dnr NL41839.098.12). The original RCT was registered in ClinicalTrials.gov with registry identifier NCT00314600. Informed consent was again obtained for participation from parents of children < 16 years and from all patients aged ≥ 12 years. This research did not receive grants from any funding agency in the public, commercial, or not-for-profit sectors. None of the authors report any conflict of interest. Completed disclosure forms for this article following the ICMJE template are available on the article page, doi: 10.2340/17453674.2023.18340

## Results

38 of 47 patients (17 of 23 with AEC and 21 of 24 with BEC) responded to the follow-up invitation and were clinically and radiologically assessed for long-term follow-up. The mean length of follow-up was 7.5 (4.4–9.6) years. Baseline characteristics were similar between the 2 groups (Table 3). In the AEC group, 2 patients were excluded for having undergone reoperation and 4 did not respond to invitation, and in the BEC group 1 patient was reoperated on and 2 did not respond to invitation (Figure). None of the patients had a re-fracture.

**Table 3:** Baseline characteristics of the population with non-reduced diaphyseal forearm fractures. Values are number of patients unless otherwise specified

Baseline	Total	AEC	BEC
Number of children	38	17	21
Age at trauma ^a^	6.4 (0.9–14.9)	6.1 (2.1–14.3)	6.7 (0.9–14.9)
Length of FU^a^	7.5 (4.4–9.6)	7.8 (5.9–9.6)	7.2 (4.4–8.8)
Male sex	18	10	8
Dominant arm	17	8	9
Type fracture radius			
Buckle fracture	0	0	0
Greenstick	27	13	14
Complete fracture	11	4	7
Type fracture ulna			
Buckle fracture	0	0	0
Greenstick	25	11	14
Complete fracture	13	6	7

AEC = above-elbow cast.

BEC = AEC with early conversion to below-elbow cast.

a Values in years are mean and (range)

**Figure:**
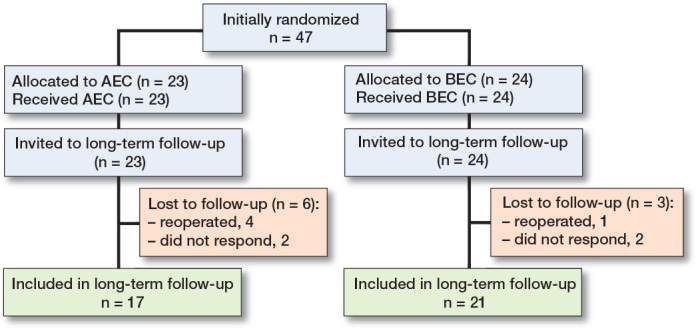
Consort patient flow diagram. Intervention was above-elbow cast (AEC) or AEC with early conversion to below-elbow cast (BEC)

Our primary outcome, loss of forearm rotation, showed improvement in both groups over time, with no significant difference between the 2 groups. The AEC group went from a mean loss of rotation of 30° (SD 29) at 2 months of follow-up, to 23° (SD 22) at 7.2 months to 8° (SD 22) at 7.5 years of follow-up. The BEC group went from 31° (SD 24) loss of forearm rotation at 2 months, to 18° (SD 17) at 7.2 months, to 8° (SD 15) at 7.5 years of follow-up. There was no significant difference between the two groups at 7.5 years of follow-up. Subgroup analyses based on the amount of loss of rotation showed no significant difference in mean limitation of prosupination (Table 4).

**Table 4:** Loss of forearm rotation of the fractured arm with subgroup analysis. Values are number of patients unless otherwise stated

Time after trauma				
Loss of forearm rotation	AEC	BEC	Mean difference (CI)	P
2 months	n = 23	n = 24		
None	3	2		
1–10°	5	3		
11–20°	3	5		
21–30°	2	5		
> 31	10	9		
Limitations ^a^	30° (29)	31° (24)	–0.9 (–17 to 15)	0.9
7.2 months	n = 23	n = 24		
None	6	6		
1–10°	4	4		
11–20°	2	6		
21–30°	3	3		
> 31°	8	4		
Limitations ^a^	23° (22)	18° (17)^b^	5.2 (–6.5 to 17)	0.4
7.5 years	n = 17	n = 21		
None	10	9		
1–10°	4	7		
11–20°	1	1		
21–30°	1	2		
> 31°	1	2		
Limitations ^a^	8° (22)	8° (15) ^b^	–0.2 (–13 to 12)	1.0

AEC = above-elbow cast. BEC = AEC with early conversion to below-elbow cast. CI = 95% confidence interval.

a Values are mean (SD)

b Significant change over time compared with the previous value in time (linear mixed-models analysis).

To address missing data, linear mixed-model analyses were performed, which showed a decrease in loss of forearm rotation over time in both groups. In the BEC group, a significant decrease was found when comparing 2 months’ loss of rotation to 7 months (P = 0.02), and 7 months to 7 years (P < 0.001).

Secondary outcomes (loss of flexion–extension of the elbow and wrist, PROMs, and grip strength) revealed no statistically significant differences between the 2 treatment groups (Table 5).

**Table 5:** Data on primary and secondary outcomes at 7.5-year long-term follow-up of non-reduced diaphyseal forearm fractures. Values are mean (SD)

Factor	AEC (n = 17)	BEC (n = 21)	Mean difference (CI)
Age at follow-up, years	13.9 (3.6)	13.9 (3.9)	0.0 (–2.5 to 2.5)
Loss of forearm rotation	5.7° (8.9)	1.5° (6.0)	4.1° (–1.5 to 9.8)
Range of forearm rotation	152° (28)	152° (18)	–0.8° (–16 to 15)
Loss of flexion–extension			
wrist	0.6° (2.5)	0° (0)	0.6° (–0.7 to 2.0)
elbow	0.0° (0)	0° (0)	/
ABILHAND-Kids score^a^	41 (3.6)	40 (7.4)	0.9 (–3.2 to 5.0)
DASH score^b^	8.2 (11)	7.6 (14)	0.6 (–8.0 to 9.2)
JAMAR ratio^c^	1.0 (0.17)	0.96 (0.19)	0.1 (–0.1 to 0.8)

AEC = above-elbow cast. BEC = AEC with early conversion to below-elbow cast. CI = 95% confidence interval.

a ABILHAND-Kids questionnaire score 0–42. 42 is optimal score.

b DASH score 0–100, 100 being the worst score.

c JAMAR ratio = grip strength affected wrist/collateral side.

At long-term follow-up we found no statistically significant differences in radiographic outcomes between the 2 groups. There was an increase in coronal radial angulation observed in both groups over time and an increase in maximal bowing (Table 6). Linear mixed-model analyses showed significant increase in the AEC group of radial coronal angulation (P < 0.001) and bowing (P < 0.001) and significant decrease in radial sagittal angulation (P = 0.001). For the BEC group only a significant decrease in sagittal radial angulation was found (P = 0.007).

**Table 6:** Data on radiographic outcomes, angulation at final follow-up. Values are mean (SD) unless otherwise stated

Factor	AEC				BEC				7.5-year mean difference (CI)
	Trauma	6 Weeks	7.2 Months	7.5 Years	Trauma	6 Weeks	7.2 Months	7.5 Years	
AP radius, ⁰	7 (4)	5 (4)	4 (4)	10 (2) ^a^	8 (6)	8 (6)	7 (6)	10 (3)	–0.2 (–1.9 to 1.6)
AP ulna, ⁰	6 (3)	5 (4)	5 (4)	5 (3)	8 (7)	6 (5)	5 (4)	4 (3)	1.2 (–0.9 to 3.3)
Lateral radius, ⁰	11 (5)	11 (5)	9 (4)	4 (2) ^a^	15 (11)	13 (7)	11 (6)	4 (3) ^a^	–0.9 (–2.9 to 1.0)
Lateral ulna, ⁰	11 (6)	5 (4) ^a^	4 (4)	4 (3)	11 (12)	7 (5)^a^	7 (5)	5 (2)	0.8 (–2.0 to 1.1)
Max. bowing radius			6 (1)	12 (2)^a^			7(3)	11(2)	0.8 (–0.6 to 2.3)

AEC = above-elbow cast. BEC = AEC with early conversion to below-elbow cast. AP = anteroposterior. CI = 95% confidence interval.

a Significant change over time compared with the previous value in time (linear mixed-models’ analysis).

We also performed a subgroup analysis comparing 2 subgroups: age < 9 years and ≥ 9 years, combining the AEC and BEC groups. Our study showed a remaining mean loss of forearm rotation of 29° in patients ≥ 9 years of age after 7.5 years, compared with 5° in children < 9 years of age (mean difference –24°, CI –38 to –9.0). There was significantly more improvement of function in patients < 9 years of age to almost no impairment. All patients, regardless of age, improved their function over time.

## Discussion

Our primary aim was to investigate whether forearm rotation improves or worsens over time. We showed that rotation improved in both groups over time, with no significant difference in the final forearm rotation: 8° (SD 22) for the AEC group and 8° (SD 15) for the BEC group with a mean difference of 0 (CI –13 to 12). Early conversion to BEC in children with a non-reduced forearm fracture of both bones is also safe at long-term follow-up of 7.5 years with both groups showing equal improvement in function with minimal persisting loss of rotation at long-term follow-up.

Our secondary aim was to analyze loss of flexion and extension of the elbow and wrist, patient-reported outcomes measures, grip strength ratio, and radiographic assessment. In accordance with the short-term follow-up, long-term follow-up of 7.5 years again showed no clinically relevant differences in either clinical or radiographic aspect between treating non-reduced minimally displaced diaphyseal both-bone forearm fractures in children with conventional AEC or with an early conversion to BEC. There were no statistically significant differences between the 2 groups for PROMs or radiographic angulations. However, it did show a significant decrease over time for radial angulation in sagittal views for both groups, which is probably due to the excellent capacity to remodel over time.

The remarkable increase in coronal radial angulation and maximal bowing after 7.5 years in both groups can be explained by the natural increase in angulation of the radius in children [8]. Firl et al. showed that the mean location of maximal bowing was 60% of the total radial length. The mean maximum radial bowing was 7.2% of the total radial length. The length of the radius and the maximum bowing increases with age, while the site of maximum radial bowing (x/y x 100) remains constant, therefore the maximum radial bowing will increase with age [9]. Another study by Weber et al. in 2019 compared 211 cadaveric specimens in humans and their mean location of maximal bowing was 45% of the radial length with a maximum radial bowing of 3.8% of the total radial length [10]. More important, however, is to know how much radial bowing is accepted before function is impaired. A recent study by Wongcharoenwatana et al. showed that children with mean radial bowing of less than 6.8% can result in < 70° forearm pronation (P = 0.03) and mean radial bowing of less than 5.8% can result in < 80° forearm supination (P = 0.02). They suggest that mean radial bowing should be restored to approximately 6–7% for optimal forearm rotation after fracture reduction. The location of the radial bowing shows no significant correlation with the amount of pro/supination limitation [11].

### Previous research

A systematic search of the literature on previous long-term follow-up studies for pediatric forearm fractures resulted in less than 15 studies in total, and none of them was an RCT. Most studies were published more than 5 years ago, and half of the studies were case reports. Therefore, our prospective randomized multi-center study presenting long-term results on diaphyseal pediatric forearm fractures is unique and an important base for substantiated statements on how to treat these fractures.

To understand why early conversion to BEC is just as good as AEC in treating pediatric both-bone forearm fractures when looking at loss of forearm rotation, it is important to understand which factors could possibly influence forearm rotation. Previous studies addressed several factors involved: malunion of the radius and/or the ulna [12-15] or contractures of the soft tissue [16]. Rotational malunion affects motion directly whereas angulation and translational malunion indirectly limits pro- and supination. Soft tissue contractures (muscle, tendon, capsule, interosseous membrane, etc.) from injury, fracture healing, and immobilization can also reduce the rotational arc of motion.

Colaris et al. showed that association of a re-fracture and a diaphyseal location are both risk factors for loss of forearm rotation with, respectively, an odds ratio of 14.1 and 2.7. Furthermore, intervention of a physiotherapist resulted in a decrease in loss of forearm rotation, which suggests that reversible soft tissue contractures might be of influence in the loss of rotation [17]. Our study showed a remaining loss of forearm rotation of 29° in patients ≥ 9 years of age. When looking at individual cases, 2 out of 5 cases ≥ 9 years had severe malunions with 85° and 47° loss of forearm rotation. The first case needed a correction osteotomy, and the second case had a bony bridge. The group < 9 years had a remaining loss of forearm rotation of 5°, with 86% (25/29 patients) improving over time. There is a significantly less loss of pro/ supination in the group < 9 years and almost all children had recovery of function over time. The degree of correction by growth is dependent on the remaining growth and the location and plane of the displacement. Early studies already demonstrated a significant relationship between age and the ability to correct deformity. Moesner and Ostergaard suggested that children under 9 years of age are able to achieve correction of 90% of their malunion and remodeling capacity decreases with age at > 9 years [18]. Högström et al. also showed that older children are less able to compensate for fracture deformities and show more remaining loss of forearm rotation [19]. It is important to know whether this remaining loss of forearm rotation had any influence in daily life. A previous study by Morrey et al. showed that a minimum of 100° of forearm rotation in total (50° of pronation and 50° of supination) is needed to perform without limitation in daily life [20]. However, later studies showed that more flexion and more pronation is necessary for daily activities because of the increased use of cellular phones and tablets.

A malunion that is located near the more active distal physis has the highest capacity to remodel. The diaphyseal part has the lowest capacity for correction [21]. Furthermore, angulation in the sagittal plane is better tolerated than angulation in the coronal plane, and rotational malunion does not remodel at all [22]. A recent study by Barvelink et al., published in 2020, showed no association between lower arm fractures with angulation and remaining motion deficit at 1-year follow-up [23]. Bot et al. in 2011 showed that functional impairment after a diaphyseal forearm fracture correlates better with subjective and psychosocial aspects of illness, such as pain and pain catastrophizing, than with objective measurements of impairment (DASH, wrist and elbow function, forearm rotation), radiographic angulation, or grip strength [3]. This is comparable to our study, which showed no relation between PROMs and the amount of loss of forearm rotation in both groups.

### Study limitations

Overall, this is a relatively favorable group, as they have little displacement with no need for reduction and, with that, they have little risk of malunion and rotation limitation. This contrasts with dislocated cases that often re-dislocate causing malunion or rotation problems, resulting in more intramedullary nailing [24].

The primary limitation was the inevitable non-blinding of the clinical assessment. Again, at long-term follow-up the initial cast morphology revealed which group the patients were in. However, radiological assessments again were blinded.

Second, this study was conducted with a small sample size of only 38 patients of the 47 primarily included patients. But with a follow-up percentage of 70%, in this young population with a relatively long follow-up period, this is a good response. Furthermore, a previous study showed that when cases are missing at random or completely at random, up to 60% loss to follow-up is acceptable, without influencing the outcome [25]. Other studies have suggested that up to 40% loss to follow-up results in minimal attrition of the results [11]. To expose any potential effects of attrition we undertook a representability analysis comparing the included and loss to follow-up groups, which showed the follow-up group was representative of the whole original study group (see Table 2).

### Conclusion

Long-term follow-up after an average of 7.5 years supports the previous suggestion in 2013 that early conversion to BEC compared with AEC as treatment for children with non-reduced diaphyseal both-bone fractures of the forearm show no significant differences between the 2 groups in loss of forearm rotation. Subgroup analyses show that children < 9 years of age have more remodeling potential. Both treatment groups show almost full remodeling, with a remaining loss of 8° of final forearm rotation and without any functional impairment in daily life. This substantiates the theory that the remaining growth behaves like a “friend” at long-term follow-up.

## Notes

LM: writing, data analysis, submitting; LD: writing, data analysis; KR: data analysis, JB: statistics; GR: reviewer, data collecting; JA: reviewer, data collecting; MR: reviewer, writing, statistics; DE: reviewer, writing; JC: reviewer, writing.

Handling co-editors: Ivan Hvid and Robin Christensen

*Acta* thanks Hanne Hedin and an anonymous reviewer for help with peer review of this manuscript.
